# Exploiting virulence determinants for precision management of melioidosis: A multi-omics roadmap from pathogenesis to point-of-care : A perspective review

**DOI:** 10.1080/21505594.2026.2732315

**Published:** 2026-09-27

**Authors:** Hua Wu, Shijia Li, Pei Zhang, Yu Su, Tao Huang

**Affiliations:** a Department of Clinical Laboratory, Hainan Women and Children’s Medical Center, Hainan Medical University, Haikou, Hainan, China; b Department of Clinical Laboratory, Hainan General Hospital, Affiliated Hospital of Hainan Medical University, Haikou, Hainan, China

**Keywords:** Melioidosis, *Burkholderia pseudomallei*, virulence factors, diagnostic biomarkers, point-of-care, multi-omics integration

## Abstract

Melioidosis, caused by *Burkholderia pseudomallei*, remains challenging to diagnose promptly, partly due to limited use of virulence factors as diagnostic targets. Most existing biomarkers fail to capture the dynamic expression of bacterial virulence factors during active infection. In vivo‑upregulated virulence determinants, such as Hcp1, POMCR/PPEP, and GroEL, are intrinsically superior diagnostic targets. Hcp1 is a paradigm: it is the most advanced virulence factor translated into a point‑of‑care test, with prospective validation in endemic settings. Mechanistic understanding of virulence regulation transforms empirical antigen hunting into rational target selection. We propose a virulence‑driven pipeline that converted Hcp1 into a point‑of‑care immunochromatography test (sensitivity 88.3%, specificity 86.1–100%) with prospective validation. Finally, multi‑omics data integration with explainable machine learning can prioritize virulence factor combinations for multiplex diagnostics. This framework addresses the need for rapid melioidosis diagnostics and provides a replicable template for other infectious diseases caused by intracellular pathogens.

## Introduction

Melioidosis, caused by the intracellular pathogen *Burkholderia pseudomallei*, remains notoriously difficult to diagnose. Current diagnostics, including culture (with reported sensitivity ranging from 50% to 70% in resource‑limited settings, requiring 3‑7 days) and polymerase chain reaction (PCR) (which requires specialized equipment and trained personnel, limiting its accessibility in many endemic regions) contributing to persistently high mortality, though patient outcomes are also influenced by comorbidities, timely antibiotic administration, and access to intensive care [1–12]. A fundamental reason for this diagnostic gap is that most biomarkers fail to capture the dynamic expression of bacterial virulence factors during active infection.

This review presents a systematic, virulence-informed framework linking *B. pseudomallei* pathogenesis to diagnostic development. The central premise, substantiated by the Hcp1 paradigm, is that virulence determinants required for bacterial survival in vivo are intrinsically enriched diagnostic targets [11,13]. We organize the evidence around three principles. First, in vivo-upregulated virulence factors are superior diagnostic targets. Second, machine learning, especially explainable AI (XAI), integrates multi-omics data into clinical decisions. Third, a four-stage translational pipeline comprising mechanistic prioritization, antigenic validation, format optimization, and field validation is reproducible. This logic is integrated into a unified visual framework (Figure 1), which charts the complete pathway from virulence factor repertoire to point of care (POC) deployment. Given Virulence’s focus on microbial pathogenicity, this review positions diagnostic development as a direct translational application of insights into *B. pseudomallei* virulence regulation. To this end, we systematically review evidence on virulence‑associated serum protein biomarkers, evaluate the translational pipeline that has converted Hcp1 into a POC test, and propose a multi-omics framework for next-generation diagnostic development. The framework comparison is summarized in Table 1.

**Figure 1.**
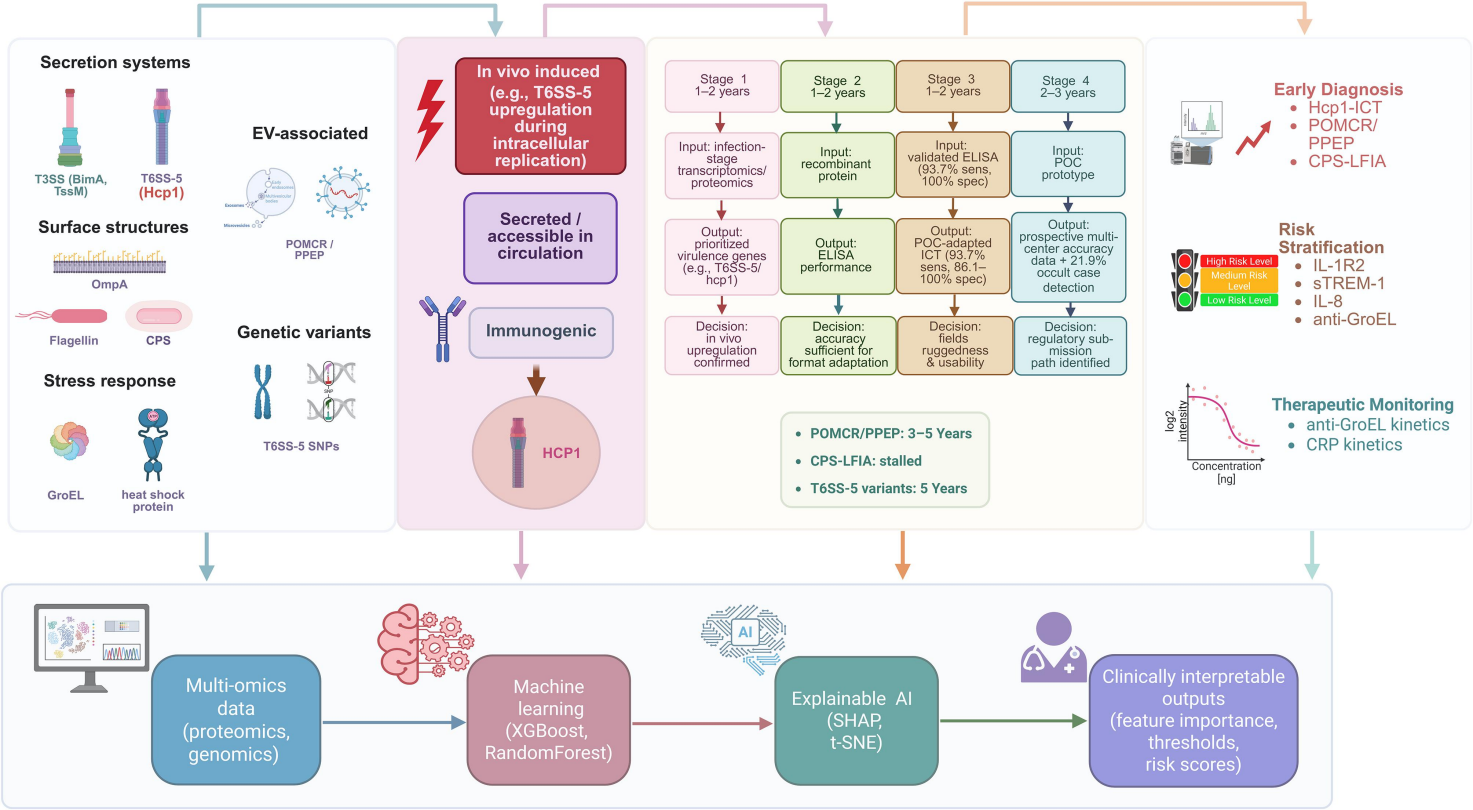
A virulence‑driven translational pipeline from pathogenesis to point‑of‑care for melioidosis.

**Left panel (Virulence factor repertoire)**: Key *B. pseudomallei* virulence determinants are organized by functional class: secretion systems (T3SS with BimA/TssM, T6SS‑5 with Hcp1), surface structures (CPS, OmpA, flagellin), stress‑response immunogen (GroEL), extracellular vesicle‑associated proteins (POMCR/PPEP), and genetic variants (T6SS‑5 SNPs informing target selection). Hcp1 is highlighted as the lead candidate.

**Middle‑left panel (Mechanism‑based prioritization)**: Three rational selection criteria: upregulated in vivo during infection (e.g. T6SS‑5/hcp1), secreted or accessible in circulation, and immunogenic, are applied to identify Hcp1 as the lead diagnostic candidate (orange‑outlined box).

**Middle‑right panel (Translational pipeline with estimated timelines)**: A four‑stage reproducible pipeline. Stage 1 (1–2 years): infection‑stage transcriptomics identifies in vivo‑induced genes (e.g. T6SS‑5/hcp1). Stage 2 (1–2 years): recombinant Hcp1 ELISA achieves 93.7% sensitivity, 100% specificity [13]. Stage 3 (1–2 years): Hcp1‑based immunochromatography test (ICT) achieves 88.3% sensitivity, 86.1–100% specificity [14]. Stage 4 (2–3 years): prospective multi‑center study demonstrates occult case detection (21.9% seropositivity in culture‑negative suspects) and ICT + TTS1‑PCR yields 98.2% sensitivity, 89.8% specificity [15]. The completed Hcp1 arc (green) required 5–7 years (2015–2023). Estimated timelines are based on the assumption that validated biomarker candidates with established ELISA platforms can progress to POC format optimization within 1–2 years, followed by prospective field validation within 2–3 years. These estimates reflect typical translational timelines observed for infectious disease diagnostics [16] and are provided for illustrative purposes rather than as definitive predictions. POMCR/PPEP (3–5 years), T6SS‑5 variants (5 years). CPS‑LFIA (prototype since 2014) is stalled (gray).

Right panel (Clinical application framework – three interconnected axes):.

Early diagnosis: Hcp1‑ICT, POMCR/PPEP, CPS‑LFIA.

Risk stratification: IL‑1R2, sTREM‑1, IL‑8, anti‑GroEL.

Treatment monitoring: anti‑GroEL kinetics, CRP kinetics.

Bottom panel (Machine learning integration): Multi‑omics data (proteomics, genomics) are integrated via machine learning (XGBoost, Random Forest) and explainable AI (SHAP, t‑SNE) to identify synergistic biomarker combinations (e.g. Hcp1 + POMCR + anti‑GroEL), establish threshold effects, and deliver clinically interpretable risk scores.

This framework exemplifies how mechanistic understanding of bacterial pathogenesis directly accelerates diagnostic translation and is extensible to other intracellular pathogens.

Abbreviations: T3SS, type III secretion system; T6SS‑5, type VI secretion system‑5; EV, extracellular vesicle; XAI, explainable artificial intelligence; ICT, immunochromatography; LFIA, lateral flow immunoassay; IL‑1R2, interleukin‑1 receptor type 2; sTREM‑1, soluble triggering receptor expressed on myeloid cells 1; CRP, C‑reactive protein.

**Table 1.** Comparison of existing melioidosis diagnostic/prognostic models with the proposed virulence-informed multi-omics framework.

Feature	Existing Models / Tools	Proposed Framework
Biomarker selection rationale	Empirical discovery; often based on convenience or literature precedent	**Mechanism-driven**: prioritizes virulence factors upregulated during active infection
Biomarker classes	Single-class (e.g. host cytokines, or pathogen antigens, or serology)	**Integrated multi-class**: pathogen-specific virulence proteins + host-response + EV-associated + humoral
Data integration method	Univariate or basic multivariate regression	**Machine learning** (XGBoost, Random Forest) with feature selection and explainable AI
Validation strategy	Single-center; retrospective	**Three-tier**: internal cross-validation + geographically distinct external cohorts + prospective POC validation
Target deployment setting	Centralized laboratories	**Tiered**: POC rapid test (peripheral) + PCR/immunoassay (regional) + proteomics (reference)
Cost profile (estimated)	Moderate to high (culture: $5–15; PCR: $20–50)	**Design-to-cost**: target <$5 per POC test
Time to result	Culture: 3–7 days; PCR: 4–6 hours	**POC: 15–30 minutes**
Quantified advantage in resource-limited settings	Not formally assessed	Reduces dependency on biosafety level 3 facilities Enables decentralized testing Identifies occult culture-negative cases (Hcp1-ICT: 21.9% seropositivity in culture-negative suspects)

## Search strategy and selection criteria

A targeted literature search of PubMed (2000–2025) was conducted using terms including “melioidosis,” “*Burkholderia pseudomallei*,” “serum proteomics,” “diagnostic biomarkers,” “machine learning,” and “POC diagnostics,” prioritizing studies with clinical validation data. We also searched the reference lists of retrieved articles. Only articles published in English were included. The final selection prioritized original research articles and reviews that directly informed the development of our integrated translational framework. This is a perspective review proposing a novel framework, and a formal systematic review or meta-analysis was not performed.

## Serum proteomics in melioidosis: from technological foundations to clinical translation

### Technological foundations and biomarker discovery

#### Overview of serum proteomics technologies in melioidosis

High-resolution mass spectrometry (LC‑MS/MS) enables the detection of low‑abundance EV‑associated proteins, but its clinical translation is hindered by cost, variability, and the need for host protein depletion [17–19]. These technical barriers underscore the urgency of developing simplified, low-cost detection formats, such as lateral flow assays, for endemic settings.

As illustrated in Figure 2, proteomic profiling has mapped an intricate regulatory network of host inflammatory, complement, and coagulation components whose dynamic changes correlate with disease severity and outcome [24–26]. However, many of these proteins (e.g. C‑reactive protein (CRP)), serum amyloid A) are nonspecifically elevated, challenging melioidosis‑specific diagnosis. Thus, combining pathogen‑specific (e.g. Hcp1) and host‑response markers (e.g. interferon‑gamma‑inducible protein 10 (IP-10)) may offer complementary value.

**Figure 2.**
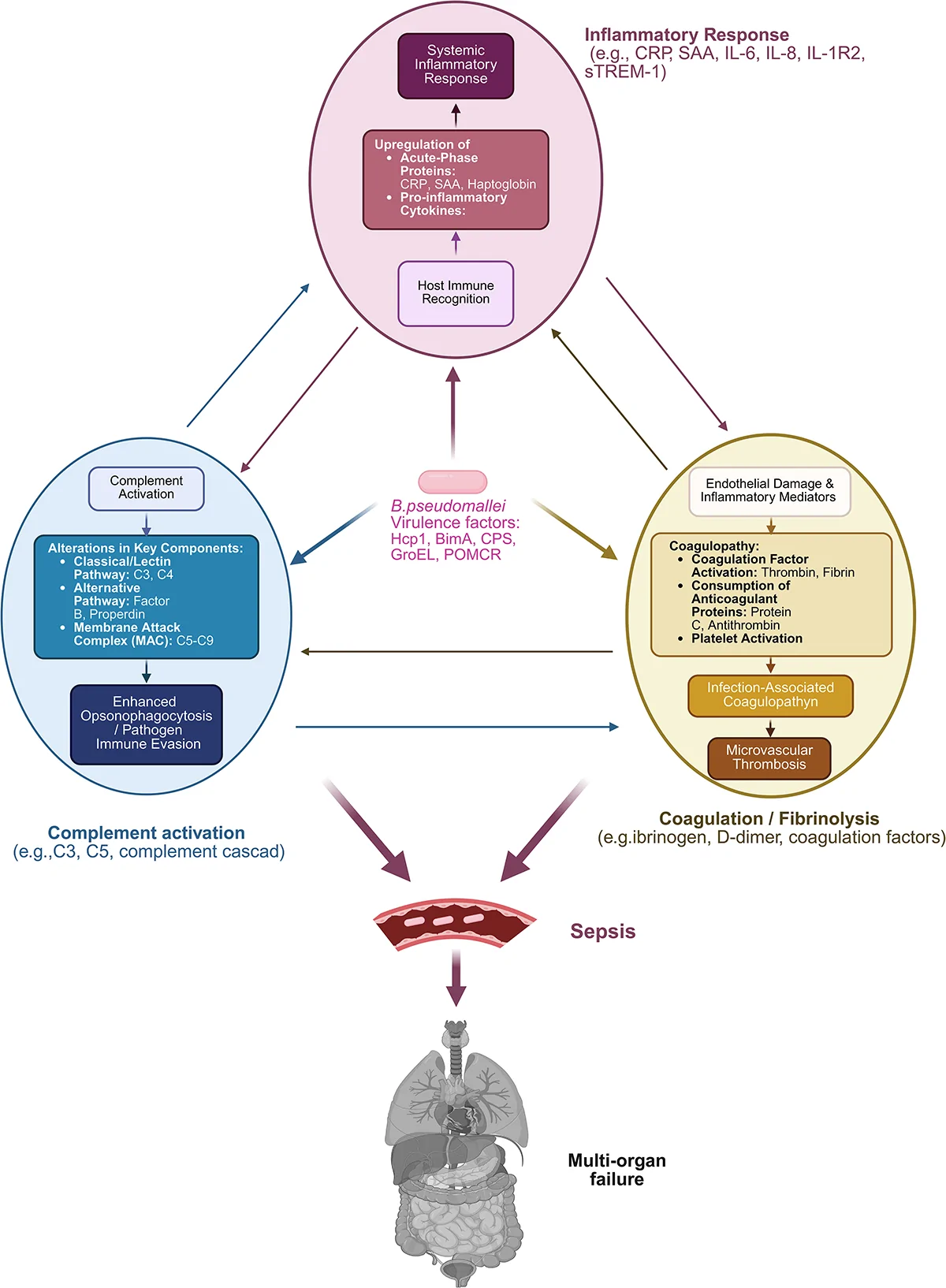
Schematic representation of key serum protein regulatory networks in melioidosis.

Proteomic profiling has mapped an intricate network of host inflammatory, complement, and coagulation components whose dynamic changes correlate with disease severity and outcome. This schematic was developed based on findings from serum proteomic studies in melioidosis and sepsis [17–23], as well as established knowledge of host inflammatory and coagulation pathways. A boxed inset on the bacterium depicts selected *B. pseudomallei* virulence factors (Hcp1, POMCR, BimA, GroEL, CPS) that trigger these host pathways. Dysregulation contributes to sepsis, which can progress to multi‑organ failure.

Bioinformatics analyses (e.g. pathway mapping) are essential to pinpoint biological processes critically perturbed during infection, shifting the focus from long lists of candidates to mechanistically grounded, virulence‑associated targets with the highest diagnostic and therapeutic utility [27–29].

Collectively, these proteomic findings establish the technological and biological foundation for virulence-informed diagnostic development. In the following section, we translate this foundation into a systematic framework for target prioritization.

## Virulence factors as diagnostic targets: from mechanistic insights to biomarker discovery

A fundamental principle underpins our framework: virulence determinants that *B. pseudomallei* upregulates during active infection are intrinsically enriched diagnostic targets. We define a “virulence‑associated diagnostic target” as a molecule that is (i) specifically upregulated in vivo during infection, (ii) encoded by a gene with a demonstrated role in pathogenesis, and (iii) accessible for non‑invasive detection. This definition prioritizes true virulence factors (e.g. T6SS‑5/Hcp1) over broadly conserved stress‑response proteins (e.g. GroEL), whose utility is primarily prognostic.

Unlike constitutively expressed antigens, these virulence‑associated proteins offer two key advantages over host‑response biomarkers (e.g. CRP, interleukin‑6 (IL‑6)): (a) etiological specificity, distinguishing *B. pseudomallei* infection from other febrile illnesses; and (b) infection‑activity correlation, as antigen levels reflect bacterial burden, whereas host proteins may remain elevated in sterile inflammation [20,21,30]. Hcp1 exemplifies this logic: its expression is driven by T6SS‑5 upregulation during intracellular replication, and its detection corresponds directly to active infection [11,13]. The overall architecture of this pipeline is summarized in Figure 1.

This pathogen-centric logic transforms our understanding of diagnostic development: instead of searching blindly for any bacterial antigen, we can rationally select targets based on their expression dynamics and functional roles during infection. The transition from a mechanistic understanding of *B. pseudomallei* pathogenesis to the development of precise diagnostic tools hinges on this premise. *B. pseudomallei* possesses an expansive arsenal of virulence factors, yet only a subset has been systematically evaluated as diagnostic biomarkers. Here, we synthesize the current evidence linking specific virulence determinants to validated or emerging diagnostic applications, with particular emphasis on those that have advanced beyond the discovery phase.

## Secretion systems as diagnostic target reservoirs

Two macromolecular secretion systems are particularly relevant as diagnostic target reservoirs in *B. pseudomallei*. The Type III secretion system (T3SS) and Type VI secretion system (T6SS) are central to *B. pseudomallei* pathogenicity. The T3SS-3 (also designated Bsa) injects effector proteins directly into host cell cytosol to modulate actin dynamics and suppress innate immunity, while the T6SS-5 is required for intracellular survival, macrophage evasion, and cell-to-cell spread [1,13]. Critically, components of these macromolecular complexes are actively synthesized during human infection and elicit robust, specific antibody responses.

*BimA* (*Burkholderia* intracellular motility A), a T3SS effector that mediates actin-based motility, has been identified as a seroreactive antigen via protein microarray screening of 1,156 *B. pseudomallei* proteins [31]. Although BimA has been evaluated in combination with other antigens in protein microarray-based serological panels, its standalone diagnostic performance requires further definition TssM, a deubiquitinase enzyme secreted via T3SS, modulates host NF-κB signaling and has demonstrated immunoreactivity in melioidosis patient sera [31]. However, its diagnostic utility relative to other antigens remains incompletely characterized.

## The T6SS-5 effector Hcp1: a paradigm of virulence-informed diagnostics

The most compelling example of a virulence factor successfully translated into a diagnostic tool is hemolysin-coregulated protein 1 (Hcp1), a component of the T6SS-5 secretion apparatus. Transcriptome profiling revealed that T6SS-5 is robustly upregulated during macrophage infection, unlike other T6SS clusters [13]. Guided by this infection-stage-specific expression pattern, Chieng and colleagues expressed three T6SS genes (*tssJ-4*, *hcp1*, *tssE-5*) as recombinant proteins and screened them against melioidosis patient sera. Only Hcp1 demonstrated immunoreactivity by both Western blot and Enzyme-Linked Immunosorbent Assay (ELISA). In a cohort of 32 culture-confirmed melioidosis patients and 20 non-melioidosis controls, recombinant Hcp1 ELISA exhibited 93.7% sensitivity and 100% specificity, substantially outperforming bacterial lysate [13].

This foundational discovery was subsequently translated into a rapid immunochromatography test (Hcp1-ICT). In a multi‑center evaluation (*n* = 487), Hcp1‑ICT achieved 88.3% sensitivity and 86.1–100% specificity, substantially outperforming IHA [14]. Prospective validation (*n* = 173) confirmed its utility and demonstrated that combining Hcp1‑ICT with TTS1‑PCR increased diagnostic accuracy to 98.2% sensitivity and 89.8% specificity, while also detecting occult melioidosis in 21.9% of initially culture‑negative patients [15].

The Hcp1 case exemplifies a complete virulence-informed translational pipeline: (i) hypothesis generation from infection-stage transcriptomics, (ii) recombinant protein expression and serological validation, (iii) format optimization into a field-deployable POC device, and (iv) prospective validation in endemic settings. This pathway provides a reproducible template for other virulence-associated candidates.

## Cell surface and secreted virulence factors

Capsular polysaccharide (CPS) is a well-established *B. pseudomallei* virulence determinant that confers resistance to phagocytosis. A prototype lateral flow immunoassay (LFIAs) targeting CPS demonstrated feasibility for rapid antigen detection [32], although sensitivity in blood specimens remains suboptimal, highlighting the complementary role of antibody-detecting formats.

Outer membrane proteins (OMPs), particularly OmpA, are conserved across *B. pseudomallei* strains, implicated in epithelial cell adhesion, and immunogenic during human infection. Recombinant OmpA-based immunoassays have been developed for veterinary melioidosis diagnosis [33] and are under evaluation as protective antigens [34].

Flagellin, the structural subunit of the bacterial flagellum, contributes to motility and TLR5-mediated immune recognition. An immunoassay incorporating recombinant flagellin and OmpA demonstrated diagnostic utility in goats with experimental melioidosis [33]; extrapolation to human diagnosis requires further validation.

## Extracellular vesicle-associated virulence factors

POMCR and PPEP are extracellular vesicle (EV)-derived proteins with exceptional diagnostic accuracy (AUC > 0.96) for distinguishing melioidosis from other febrile illnesses [17]. Although their virulence functions are unknown, their specificity and EV association suggest they are pathogen-secreted products linked to bacterial fitness.

In addition to EV‑associated proteins, stress‑response immunogens such as GroEL have also been investigated as biomarkers, though their role is primarily prognostic rather than diagnostic.

## Emerging virulence-associated targets

Recent multi‑omics investigations have expanded the repertoire of candidate virulence‑associated biomarkers. Previous genomic studies have demonstrated that large‑scale deletions encompassing the T6SS‑5 cluster impair intracellular survival and plaque‑forming efficiency in environmental *B. pseudomallei* isolates [28]. Collectively, these data suggest that T6SS‑5 genetic integrity, at both the presence/absence and sequence polymorphism levels, may influence bacterial virulence and, consequently, disease severity. Although such genetic variants are not themselves circulating biomarkers, they inform target antigen selection by highlighting virulence determinants under diversifying selection during human infection. Similarly, integrated proteogenomic analyses of host‑pathogen protein interaction networks have predicted novel *B. pseudomallei* effectors that interact with human immune regulatory proteins [27], providing a computationally prioritized pipeline for biomarker discovery.

## Critical perspective

While Hcp1-ICT has shown promise in Thai cohorts, its performance in other endemic regions remains untested. Similarly, POMCR/PPEP require multi-center validation before POC translation. Antibody persistence may limit utility in high-exposure settings.

Having established the mechanistic rationale for virulence-informed target selection, we now examine how these targets can be systematically validated and integrated into multi-biomarker diagnostic models.

## Serum proteomics in the diagnosis of melioidosis

The biological complexity of melioidosis contrasts with limitations of current diagnostic methodologies. Diagnosis remains challenging due to constraints of conventional approaches.

The clinical imperative: shortcomings of the current diagnostic armamentarium. Diagnosing melioidosis is challenging due to conventional methods’ constraints. Culture-based approaches, though the gold standard, exhibit limited sensitivity and require prolonged incubation (3–7 days). Serological assays (e.g. IHA, ELISA) are hampered by cross-reactivity, reducing specificity. Molecular techniques like PCR improve accuracy but entail high costs and infrastructure demands, limiting scalability in resource-limited settings.

Emerging technologies such as single-cell proteomics enable granular immune response analysis but face challenges including limited multiplexing capacity, high costs, and analytical complexity [29,35]. Serum proteomics offers broader proteome-wide screening but lacks cellular resolution [35].

Clinically, nonspecific presentation and diagnostic limitations often result in missed or delayed diagnosis, contributing to high mortality. Thus, scalable, rapid, and accurate tools are urgently needed. Serum proteomics represents a promising strategy but requires standardization, cost reduction, and validation in diverse cohorts [36,37].

## Screening and validation of serum protein biomarkers

### The biomarker translation pipeline: from discovery to validation

Converting a proteomic discovery into a validated diagnostic tool requires a rigorous pipeline that distinguishes true signals from biological noise. This process begins with high-throughput screening to identify candidate biomarkers, followed by a multi-stage validation protocol to confirm their diagnostic utility.

Proteomics provides a powerful approach for identifying serum protein biomarkers, enabling detection of pathogen-specific antigens or host-derived inflammatory mediators. High-throughput screening techniques, such as mass spectrometry and protein microarrays, have uncovered numerous candidates. For instance, protein microarrays identified *Mycobacterium tuberculosis* antigen panels with high sensitivity and specificity, a strategy applicable to melioidosis [36].

Promising candidates must then undergo rigorous validation, typically via immunoassays like ELISA, to quantify their differential expression and diagnostic performance (sensitivity, specificity). Given the pathological heterogeneity of melioidosis, combining virulence-associated pathogen-specific antigens (e.g. Hcp1, OmpA, POMCR/PPEP) with host-response markers into multi-marker panels is a key strategy to achieve the required diagnostic robustness, a principle successfully applied in other complex diseases like cancer [38,39].

Further validation across diverse clinical cohorts and disease stages is essential [36,37] to establish the generalizability of promising biomarkers such as the EV-derived proteins POMCR and PPEP, which have shown exceptional accuracy in distinguishing melioidosis from other febrile illnesses in initial studies [17]. However, translating these biomarkers into clinical practice faces major barriers: limited generalizability due to small, region-specific cohorts [40]; high costs and technological reliance on methods like mass spectrometry, necessitating affordable alternatives (e.g. lateral flow assays) [41,42]; and unresolved diagnostic specificity, as biomarkers must be validated against mimics such as tuberculosis and dengue, exemplified by host protein p62/NBR1 requiring further investigation [43].

To advance these biomarkers toward clinical utility, future research must: (1) prioritize multi-center prospective cohorts with standardized protocols; (2) validate diagnostic thresholds across different clinical presentations; and (3) conduct adaptability testing for POC to integrate promising biomarkers (e.g. Hcp1 [13–15], POMCR/PPEP [17]) into rapid, low-cost formats. This requires addressing pre-analytical variables (e.g. hemolysis effects) and assessing technical feasibility in field conditions.

In summary, the journey from biomarker discovery to clinical impact is a multi-stage translational pathway from Discovery through high-throughput screening, to Verification & Panel Optimization using targeted assays and machine learning, culminating in Clinical Implementation through POC devices. The path to implementation is fraught with challenges, including generalizability, cost, and specificity, that represent major barriers at each stage [32,44,45]. A systematic approach is paramount for converting proteomic promises into practical diagnostic solutions.

## Construction of multi-biomarker diagnostic models for melioidosis

The pathological heterogeneity of melioidosis necessitates diagnostic models that move beyond single biomarkers. To this end, machine learning is employed to intelligently integrate the diverse classes of serum proteins (pathogen-specific virulence factors, host-response, EV-derived, and humoral immune indicators, as detailed in Table 2) into a unified diagnostic signature. These models are designed to capture the complex, non-linear interactions among biomarkers, thereby constructing a robust diagnostic tool that accounts for the variability in both host response and pathogen expression, ultimately aiming for superior accuracy across diverse patient populations [17,20–23,31,47]. Beyond a simple positive/negative result, such a model could provide a probability score to directly support clinical decision-making at the POC.

**Table 2.** Virulence-associated diagnostic targets for melioidosis: mechanisms, evidence, and translational readiness.

Virulence Factor/Antigen	Biological Function/Virulence Mechanism	Diagnostic Format	Performance Metrics	Translational Stage	Main Limitation(s)	Key References
Hcp1	T6SS-5 effector; required for intracellular survival and macrophage evasion	ELISA,Immunochromatography (ICT)	ELISA: 93.7% sens, 100% spec (*n* = 52) ICT: 88.3% sens, 86.1–100% spec (n = 487) ICT + TTS1-PCR: 98.2% sens, 89.8% spec (n = 173)	WHO-prequalifiable POC test (prospectively validated)	Antibody persistence (may limit utility in high-exposure settings)	[13–15]
Capsularpolysaccharide (CPS)	Anti-phagocytic; essential for virulence	Lateral flow immunoassay (antigen detection)	Prototype developed; limited sensitivity in blood	Prototype phase	Low sensitivity in blood	[32]
POMCR/ PPEP	EV-associated proteins; function under investigation	LC-MS/MS discovery; ELISA prototype	AUC > 0.96 (discovery cohort)	Discovery / early validation	Single-center, unvalidated	[17]
*BimA*	T3SS effector; actin-based intracellular motility	Protein microarray; serological panel	Immunoreactive; diagnostic performance under evaluation	Panel validation	Standalone performance undefined	[31]
TssM	T3SS-secreted deubiquitinase; NF-κB modulation	Protein microarray	Immunoreactive; standalone performance undefined	Candidate identification	Diagnostic utility undefined	[31]
OmpA	Outer membrane protein; epithelial adhesion	ELISA (veterinary)	Goat melioidosis diagnosis	Cross-species validation	Veterinary only (human data lacking)	[33,34]
GroEL	Heat shock protein; intracellular stress response	ELISA	Caprine: correlates with clearance Human: anti-GroEL IgG2 associated with survival	Prognostic/monitoring biomarker (not for diagnosis)	Not pathogen‑specific; diagnostic use not recommended	[20,46]
Flagellin	Motility; TLR5 ligand	ELISA (veterinary, with OmpA)	Diagnostic in experimental goat infection	Veterinary application	Veterinary only (human data lacking)	[33]

To ensure model robustness and generalizability, we recommend a three-tier validation strategy with explicit sample size justification and overfitting countermeasures:

1. Internal validation: k-fold cross-validation (k = 5 or 10) with stratification by disease status and key covariates (diabetes, bacteremia). Minimum sample size for discovery cohorts should be guided by the events-per-variable (EPV) rule, with EPV ≥ 10 for binary outcomes. For a panel of 10–15 candidate biomarkers, a minimum of 150–225 melioidosis cases is recommended.
2. External validation: Independent validation in at least two geographically and ethnically distinct cohorts (e.g. Northeast Thailand, Northern Australia, Indian subcontinent) to assess transportability. Performance heterogeneity should be quantified using meta-analytic methods (I^2^ statistics).
3. Prospective validation: Real-world evaluation in peripheral district hospitals and primary care clinics in endemic regions, with intentionally varied pre-analytical conditions (delayed centrifugation, ambient temperature storage, hemolyzed/lipemic samples) to reflect field realities.

Overfitting control should be embedded at multiple levels: (i) feature selection using regularized regression (LASSO, elastic net) or tree-based feature importance with cross-validated pruning; (ii) nested cross-validation for model tuning and performance estimation; (iii) independent test set held out from all model development steps.

Critical validation gaps (Table 2) must be addressed. No multi-biomarker diagnostic for melioidosis has received FDA or CE certification, highlighting the implementation gap (Figure 3).

**Figure 3.**
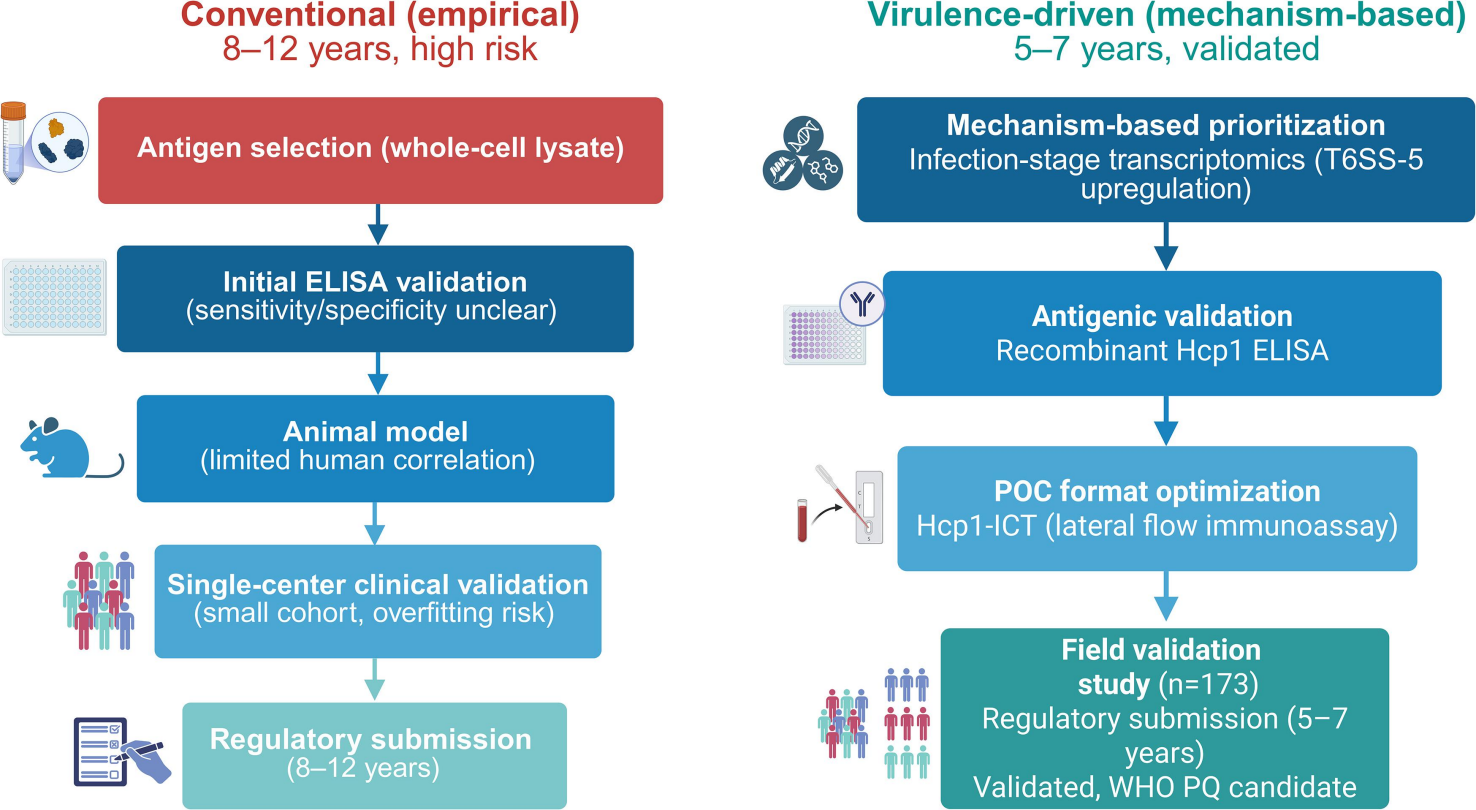
Comparative translational pathways for melioidosis biomarker development.

**Left (Conventional empirical pipeline)**: Antigen selection (whole‑cell lysate), followed by initial ELISA validation, then animal model, then single‑center clinical validation, and finally regulatory submission (8–12 years). This pathway carries a high risk of failure.

**Right (Virulence‑driven mechanism‑based pipeline exemplified by Hcp1)**: Mechanism‑based prioritization (infection‑stage transcriptomics, T6SS‑5 upregulation), followed by antigenic validation (recombinant Hcp1 ELISA: 93.7% sensitivity, 100% specificity), then POC format optimization (Hcp1-ICT: 88.3% sensitivity, 86.1–100% specificity), and finally field validation (prospective study, *n* = 173: 21.9% occult case detection, 98.2% sensitivity/89.8% specificity when combined with TTS1-PCR). The pipeline was completed in 5–7 years and is a WHO PQ candidate.

Key performance metrics are highlighted. The right pathway is colour‑coded green/orange to indicate success.

Translation requires formal cost-benefit analysis. While development and validation need significant investment, potential to reduce mortality through early diagnosis could lead to substantial long-term savings by decreasing hospitalizations, ICU admissions, and inappropriate antibiotic use. Future health economic studies should evaluate cost-effectiveness in resource-limited settings.

## Machine learning and multi‑omics integration

The integration of machine learning with multi‑omics data offers a promising opportunity to move toward a mechanistic understanding of melioidosis pathophysiology [27]. In the absence of large, publicly available datasets, we present a conceptual framework illustrating how explainable AI (XAI) could be applied. The following analysis is based on simulated data and is intended to demonstrate the analytical workflow, not to report validated model performance.

Decoding systems‑level patterns provides a biological rationale for biomarker selection, ensuring signatures are causally grounded. Machine learning offers three distinct advantages: capturing non‑linear interactions (differentiating melioidosis from other febrile illnesses), identifying threshold effects, and selecting robust features via regularized regression.

Model interpretability: building clinician trust. Explainable AI (XAI) transforms “black box” outputs into transparent, verifiable decisions. In our illustrative analysis, SHAP reveals that Hcp1 seropositivity (31%), POMCR (22%), and anti‑GroEL (18%) drive model predictions. A physician can immediately see which biomarkers drove a risk prediction, enabling confidence in rule‑out/in thresholds, critical in melioidosis where empirical treatment is toxic and diagnosis is often delayed [1,5]. Supplementary Figure 1 illustrates how t‑SNE projection and SHAP dependence plots can inform the design of binary algorithms for POC implementation.

(Note: This analysis is illustrative; actual implementation requires empirical multi‑omics data.)

## Prognostic assessment and therapeutic monitoring for melioidosis

### Identification of prognostic-related proteins

Identifying serum proteins linked to disease severity, treatment response, and survival is crucial for improving prognostic assessment. Serum proteomics captures systemic alterations, with inflammatory mediators, immune regulators, and metabolism-related proteins emerging as valuable prognostic indicators.

In melioidosis, the prognostic potential of the host immune response is exemplified by anti-GroEL antibody kinetics, which correlate with survival outcomes in experimental models [20]. This suggests inflammatory mediators, complement components, and tissue repair proteins are strong candidate biomarkers awaiting clinical validation.

GroEL: A prognostic marker for treatment monitoring. GroEL is a highly conserved heat shock protein, but its homologs in other bacteria and human mitochondria limit its specificity as a diagnostic marker for active melioidosis. In a caprine model, anti‑GroEL IgG kinetics correlated with bacterial clearance and survival [20], and GroEL is a major antibody target in human melioidosis [48]. Elevated levels of *B. pseudomallei*‑specific antibodies, including those targeting GroEL, are associated with reduced mortality in humans [46]. Therefore, we position GroEL as a complementary prognostic and treatment‑monitoring biomarker. Its value lies in serial measurement during therapy, where changing antibody titers may reflect treatment response or relapse risk. However, large‑scale human validation is lacking, and its specificity against other infections remains untested, making it a priority for prospective cohort studies.

## Development of prognostic prediction models

Validated multi-parameter prognostic models for melioidosis are still lacking, but several promising biomarker classes have emerged [27]. Building robust prognostic prediction models based on serum protein expression profiles represents a promising avenue for enhancing clinical decision-making in infectious diseases with complex pathophysiology. Approaches employing multivariate analyses and machine learning algorithms have enabled the construction of prognostic scoring systems that integrate multiple protein biomarkers to predict disease outcomes.

In the domain of diagnosis, one of the most promising advances involves the discovery of extracellular vesicle (EV)-derived protein biomarkers. A study focusing on the proteins POMCR and PPEP reported exceptional diagnostic accuracy, with AUC greater than 0.96 for distinguishing melioidosis from other infections [17], suggesting high potential for developing a highly sensitive immunoassay. However, this model requires validation in larger, multi-center cohorts to confirm its generalizability.

For prognostic assessment, the host humoral immune response has shown significant value. Developing such prognostic models is particularly crucial for melioidosis given its heterogeneous outcomes. Research has demonstrated that high levels of anti-GroEL IgG are strongly associated with improved survival in melioidosis patients [48]. While this identifies anti-GroEL as a robust prognostic indicator, its utility as a standalone factor or its integration into a multi-variable prognostic model for clinical use requires further prospective validation. Technical proof-of-concept for such models exists in other diseases. For example, in intracranial aneurysm, logistic regression models incorporating proteomic data can differentiate disease states and predict rupture risk [49]. For melioidosis, the immediate imperative is to leverage robust candidate biomarkers (e.g. anti-GroEL) to build and validate analogous multivariate models, tailored to its unique pathophysiology. In multiple sclerosis, a multivariate proteomic disease activity (MSDA) test has been analytically and clinically validated to reflect disease activity and treatment response, demonstrating the utility of proteomic-based scoring systems in chronic inflammatory conditions [50].

These models not only provide prognostic information but also support clinical decisions such as tailoring treatment regimens and monitoring relapse risk. For example, a model that identifies patients at high risk of fatal outcomes could prompt earlier ICU admission and more aggressive therapeutic interventions, thereby optimizing resource allocation in busy endemic hospitals. The dynamic nature of the serum proteome facilitates longitudinal monitoring, enhancing the predictive power of these models. Incorporating proteomic data into clinical workflows can thus enable personalized medicine approaches, optimizing therapeutic efficacy and minimizing adverse outcomes.

In summary, the current landscape is defined by high-potential biomarker discoveries rather than mature clinical models. The important next step for the field is to integrate these promising biomarkers into multivariate frameworks using machine learning and to validate them prospectively across diverse endemic settings to bridge the gap between discovery and clinical impact.

## Application of serum proteomics in monitoring treatment response

**S**erum proteomics enables monitoring of treatment response in melioidosis by tracking dynamic protein expression changes that reflect therapeutic efficacy. Longitudinal analyses show that protein level changes during treatment correlate with biological responses and clinical outcomes. For instance, antibody levels against *B. pseudomallei* antigens such as GroEL fluctuate during infection and therapy. Translating this from model to clinic is paramount. In a goat model, anti-GroEL IgG responses correlated with bacterial clearance [20], suggesting a potential role in monitoring therapy. This mirrors the critical importance of appropriate antibiotic therapy in human melioidosis, where inappropriate empiric regimens are significantly associated with mortality [44]. However, human data on serial anti-GroEL kinetics are currently lacking. Similarly, decreases in host inflammatory proteins (e.g. CRP) or pathogen-specific proteins may indicate treatment success, whereas persistent elevation could suggest resistance or chronic infection.

## Critical perspective

While animal models provide initial insights, human data on treatment monitoring biomarkers remain limited. The specificity of these biomarkers for melioidosis vs other infections requires further investigation, and the absence of standardized cutoff values for protein changes hinders clinical implementation. Future studies should prioritize longitudinal human samples to establish reliable monitoring protocols.

The minimally invasive nature of serum sampling allows repeated measurements, supporting real-time therapy assessment. Thus, serum proteomics holds significant potential for personalized treatment monitoring in melioidosis through the tracking of protein kinetics. Therefore, a proteomic-guided monitoring tool could transform therapy. Serial measurement of key biomarkers has the potential to guide the duration of intravenous and oral eradication therapy, directly addressing the challenge of relapse (reported in 5–13% of cases, often linked to premature cessation or suboptimal treatment) and helping to avoid unnecessarily prolonged hospitalization (mean ~51 days [45]).

In summary, serum proteomics offers a powerful means for evaluating therapeutic efficacy and providing an early warning of relapse by tracking the kinetics of antibodies (e.g. anti-GroEL) and host inflammatory proteins (e.g. CRP) during treatment. As summarized in Figure 1, dynamic serum proteomic data forms the core of an integrated clinical management framework, with its utility manifesting across three interconnected axes: achieving early and accurate diagnosis, enabling personalized risk stratification, and facilitating real-time treatment monitoring. This framework systematically illustrates how proteomic analysis can be translated into decision-support information throughout the entire patient management journey.

## Clinical translation and future perspectives

Building upon the integrated framework outlined in previous sections, the translation of proteomic discoveries into clinical practice represents the critical next step in melioidosis management. Serum proteomics has evolved significantly from basic research to clinical applications, offering paradigm-shifting potential for substantial melioidosis diagnosis and patient stratification. This translation is driven by advances in mass spectrometry (MS)-based methods, which enable highly specific detection of disease-specific protein biomarkers directly from serum samples. For instance, recent studies have identified highly specific biomarker signatures for melioidosis, such as extracellular vesicle (EV)-derived proteins (e.g. POMCR and PPEP), which demonstrated exceptional diagnostic accuracy in distinguishing melioidosis from other infections [17]. These findings highlight the potential for rapid, noninvasive assays tailored to melioidosis.

The paramount challenge is not discovery, but translation into clinically actionable, accessible, and affordable tools for resource-limited endemic regions. This complexity is underscored by cases of disseminated melioidosis with septic arthritis being misdiagnosed as tuberculosis due to diagnostic limitations [51], highlighting the urgent need for better solutions. To overcome this, targeted proteomics approaches (e.g. selected reaction monitoring) can be applied to validate predefined protein panels, such as those involving *B. pseudomallei* outer membrane proteins (e.g. OmpA) that have been implicated in virulence and immune response [34]. Integration with proteogenomics has further refined biomarker specificity by elucidating host-pathogen interactions, as predicted in studies mapping *B. pseudomallei-*human protein networks [27].

As detailed in the previous section and summarized in Table 2, Hcp1‑ICT has been prospectively validated in over 650 patients [14,15].

## Beyond lateral flow: emerging POC platforms

Microfluidic and smartphone‑based readers could enable multiplexed detection of pathogen‑specific (Hcp1, POMCR) and host‑response (anti‑GroEL, IL-1R2, sTREM-1) biomarkers. However, translation requires design‑to‑cost trade‑offs, as multiplexing increases cost and demands stable power and connectivity not always available in remote areas [32]. A tiered solution using single‑analyte Hcp1‑ICT for peripheral screening and multiplex platforms for reference laboratories may be optimal.

Systematic challenges and solutions. The transition from discovery to field deployment faces substantial hurdles. Validation studies are often confined to optimized research settings, failing to reflect endemic realities. A summary of these translational challenges and strategic solutions is presented in Table 3.

**Table 3.** Key challenges and proposed solutions for translating serum proteomics and virulence-informed diagnostics into melioidosis clinical practice.

Challenge	Proposed Solutions / Future Directions
1. Generalizability and Validation	Establish an international melioidosis proteome consortium for multi-center, prospective studies. Validate diagnostic thresholds and prognostic models in geographically and ethnically diverse populations.
2. Technological and Economic Barriers	Develop low-cost, ruggedized alternatives such as Lateral Flow Immunoassays (LFIA) targeting signature biomarkers (e.g. Hcp1, POMCR/PPEP, GroEL). Invest in miniaturized and portable detection systems.
3. Standardization and Reproducibility	Define and disseminate Standard Operating Procedures (SOPs) via international consortium. Systematically evaluate and address pre-analytical variables (hemolysis, temperature) under field conditions.
4. Translational Gap	Prioritize real-world validation studies in endemic field hospitals and primary care settings Adopt a design-to-cost approach for POC device development from the outset.
5. Data Integration and Interpretability	Apply Explainable AI (XAI) techniques (SHAP, t‑SNE) as integrated components of the proposed framework. Build larger, well-annotated datasets specifically for melioidosis to train more robust models. Establish international consortium for multi-center validation and data harmonization, addressing underpowered cohorts and lack of standardized data with transparent reporting of negative results.

Technology Co-Development: Parallel to biomarker validation, there must be a dedicated effort to co-develop simplified, ruggedized assay formats. Success hinges on multidisciplinary collaboration between proteomic scientists, clinical microbiologists, and biomedical engineers to tailor innovations to real-world constraints.

To translate these discoveries into practice, an algorithm-driven implementation pathway is essential. The overarching framework (Figure 1) positions this Hcp1 example within a broader, generalizable pipeline. This begins with technical validation of a multi-biomarker panel defined by machine learning. The validated model can initially be deployed as a cloud-based diagnostic tool integrated into Laboratory Information Systems in endemic region hospitals. The ultimate goal is a digitally connected, low-cost POC device utilizing a microfluidic immunoassay to detect the biomarker panel, with a built-in processor to run the diagnostic algorithm on-site, delivering results within minutes. Prototype lateral flow immunoassays targeting *B. pseudomallei* capsular polysaccharide demonstrate this approach’s feasibility [32].

## Discussion

The clinical utility of the proposed pipeline stems from its foundation in pathogen biology: each diagnostic target was selected based on its in vivo expression pattern and virulence function.

## From virulence mechanism to bedside: a reproducible translational pipeline

The Hcp1 paradigm exemplifies a reproducible four-stage translational pipeline, including mechanistic prioritization, antigenic validation, format optimization, and field validation, that has successfully converted an in vivo-induced virulence factor into a validated POC diagnostic tool [13–15]. This pipeline is now being extended to other targets (POMCR, GroEL, T6SS-5 variants) and is broadly applicable to neglected tropical diseases where infection-induced antigens can be distinguished from environmental exposures.

## Putting this framework in context

Unlike prior reviews that catalog biomarkers without a translational roadmap, this work uniquely integrates virulence-informed target prioritization, multi-omics, and POC engineering into an actionable framework for endemic settings.

## Comparative proteomics: lessons from tuberculosis and sepsis

Two specific lessons emerge from tuberculosis and sepsis. First, TB serodiagnosis has successfully employed multi‑antigen panels incorporating stage‑specific immunogens (ESAT‑6, CFP‑10) to distinguish active infection from latent infection or prior BCG vaccination [36]. This approach directly parallels our strategy of prioritizing infection‑upregulated virulence factors (T6SS‑5/Hcp1, POMCR/PPEP) over constitutively expressed antigens. Importantly, the TB experience offers a cautionary as well as instructive example: while multi-antigen panels improved specificity, they did not eliminate the fundamental challenge of distinguishing active from past infection. An alternative approach to this challenge is to prioritize antigens that are not only specific but also dynamically upregulated during active infection, a strategy that has been successfully applied to Hcp1 in melioidosis. Second, sepsis management has integrated host‑response proteins (procalcitonin, CRP, IL‑6) into clinical algorithms for antibiotic stewardship. In melioidosis, similar host markers have shown prognostic value. IL‑1R2, sTREM‑1, and IL‑8 are associated with mortality risk [20,22], and elevated levels of *B. pseudomallei*‑specific IgG2 are associated with improved survival [46]. Notably, GroEL is a major target of the antibody response in melioidosis [48], suggesting that anti‑GroEL antibodies may contribute to this protective humoral immunity. The unique opportunity lies in combining host biomarkers with pathogen‑specific virulence markers, a hybrid strategy that neither TB nor generic sepsis can exploit.

While the present framework draws lessons from TB’s antigen selection strategy, it also recognizes that the hybrid pathogen‑host biomarker approach developed for melioidosis could, in turn, inform TB diagnostics by integrating host biomarkers (e.g. IP‑10) with stage‑specific antigens. This bidirectional learning opportunity underscores the generalizability of the framework: the principles of virulence‑informed target prioritization, multi‑omics integration, and POC translation are not disease‑specific. They can be adapted to any intracellular pathogen where infection‑upregulated antigens can be distinguished from constitutively expressed proteins.

## The unique advantage of a “pathogen-host hybrid” strategy

Critically, melioidosis occupies a privileged position: it is one of few infectious diseases where both pathogen-specific virulence factors and host-response prognostic markers have been rigorously validated in parallel. Tuberculosis has ESAT-6/CFP-10 but no equivalent host marker for outcome prediction; sepsis has PCT/IL-6 but no pathogen-specific target. Our framework’s integration of Hcp1 (pathogen-active) with anti-GroEL (host-prognostic) into a single algorithmic model is therefore conceptually novel and could serve as a template for other neglected tropical diseases with similar diagnostic gaps.

Furthermore, our framework’s use of machine learning for multi-omics integration shows proven potential. The validation of a multivariate proteomic test for multiple sclerosis [50] provides a template for melioidosis, demonstrating that complex protein signatures can become clinical tools for treatment monitoring.

Thus, by systematically linking bacterial virulence regulation to diagnostic target prioritization, this framework not only advances melioidosis management but also offers a paradigm that other researchers studying other intracellular pathogens such as *Mycobacterium tuberculosis* or *Salmonella Typhi* could directly adopt.

### A bidirectional bridge between pathogenesis and diagnostics

Importantly, the successful translation of Hcp1 into a POC test also informs our understanding of *B. pseudomallei* pathogenesis. The observation that anti‑Hcp1 antibodies are detectable only during active infection provides indirect clinical evidence that T6SS‑5 is indeed upregulated in human disease, a finding originally derived from macrophage infection models [13]. This bidirectional bridge, from mechanistic insight to clinical application and back, represents a core value proposition of our framework. Diagnostic development serves not only as an endpoint but also as a validation tool for virulence hypotheses.

## Ethical and health economic imperatives for translation

The translation of proteomic discoveries is an ethical and economic necessity for resource-limited populations bearing the highest burden of melioidosis. Achieving equity in diagnostic access demands more than technological innovation; it requires a fundamental re-orientation toward “design-to-cost” and “design-for-field” principles. This means prioritizing the development of affordable, rugged platforms like LFIAs from the outset, rather than adapting complex laboratory technologies [32].

## Cost composition and supply chain considerations

The unit cost of Hcp1-ICT is estimated at $2‑$3 (USD) per test, compared to 10–20 for bacterial culture (excluding infrastructure and personnel). The Hcp1‑ICT format offers several additional advantages over culture and PCR: it requires no cold chain (stable at 4‑30°C for over 12 months), produces results within 30 minutes, and can be performed without specialized equipment. In contrast, PCR reagents require cold storage (−20°C) and culture media require refrigeration (2‑8°C), limiting their use in regions with unreliable electricity. Innovative pricing and local manufacturing strategies could further enhance affordability and accessibility in resource-limited settings [30].

A health economic framework can quantify this advantage. The following scenario is a simplified illustrative model based on published estimates of diagnostic costs, antibiotic therapy, and ICU stay in Southeast Asian settings [44,45,52]. Assume a district hospital in Northeast Thailand with 50 suspected melioidosis admissions per year, of whom 20 are culture-positive. Under current standard care: culture costs $10 per test (3–7 days), empirical ceftazidime for 14 days costs $200 per patient, and the average ICU stay for septic patients is 5 days at $500 per day. The total annual cost for 20 confirmed cases is approximately (20 ×$10) + (20 ×$200) + (10 ICU-bound patients ×$500 × 5) = $200 + $4,000 + $25,000 = $33,200, not accounting for prolonged hospitalization due to relapse (reported in 5–13% of cases) [44,45].

An alternative POC-based algorithm using Hcp1-ICT ($3 per test, 30-minute result) enables same-day targeted therapy initiation. ICU stay is reduced from 5 to 2 days for those testing positive [53], and empiric antibiotics are avoided in negatives [52]. The revised annual cost is approximately (20 ×$3) + (20 ×$200) + (10 ICU-bound patients ×$500 × 2) = $60 + $4,000 + $10,000 = $14,060, a saving of more than 50%. This simplified model excludes averted relapse costs and productivity losses; rigorous cost-effectiveness analyses are urgently needed.

## Future directions: beyond discovery to implementation

### Limitations and unresolved questions

Several limitations of the proposed framework warrant consideration. First, the sensitivity of virulence‑informed serological tests may be compromised during early or low‑burden infection stages, when bacterial replication and virulence gene expression are minimal. Second, environmental exposure to *B. pseudomallei* can elicit low‑level antibodies without causing clinical disease, potentially confounding serological interpretation even with specific antigens such as Hcp1. The extent to which background seropositivity in highly endemic populations affects diagnostic specificity remains to be quantified. Third, genetic and antigenic variation among *B. pseudomallei* strains from different geographic regions may influence the performance of Hcp1‑ICT and other antibody‑based tests. Prospective validation in Australia, India, and Brazil is urgently needed. Fourth, the machine learning component of our framework is presently conceptual; real‑world application requires large, standardized multi‑omics datasets that are not yet available. We hope this framework will catalyze efforts to generate such data. Finally, while we advocate for multi‑analyte POC devices, the optimal combination of biomarkers (e.g. Hcp1, POMCR, anti‑GroEL) and the relative weight of pathogen‑specific vs host‑response markers in diagnostic algorithms require empirical optimization.

Beyond scientific and technical challenges, practical barriers to clinical adoption warrant consideration. These include regulatory approval timelines, which typically span several years even for well‑validated diagnostic tests; assay standardization across different manufacturers and lot numbers; and accessibility in diverse endemic regions, where infrastructure and supply chains vary substantially. Addressing these implementation barriers will require early engagement with regulatory agencies, collaboration with local manufacturers, and context‑specific health economic evaluations.

Looking forward, we identify three priority research and development axes that will determine whether the proposed framework achieves its intended public health impact.

### Expansion of the virulence-informed biomarker pipeline

While Hcp1 serves as a proof-of-concept, additional T6SS-5 components (e.g. VgrG-5, PAAR proteins) and other in vivo-induced antigens merit systematic evaluation. Emerging proteomic technologies (e.g. immunopeptidomics, virion capture) could accelerate the discovery of secreted virulence factors directly accessible in patient biofluids.

### Integration of digital health technologies

The diagnostic algorithms developed through our framework are intrinsically suited for deployment as clinical decision support systems embedded within electronic health records. In parallel, smartphone-based lateral flow readers and AI-assisted interpretation apps can extend POC testing to the lowest levels of the health system. Machine learning models could be continuously updated with real-world data to refine diagnostic thresholds and adapt to shifting epidemiology.

### Regulatory and commercial pathways

To achieve WHO prequalification and widespread adoption, biomarker validation must adhere to rigorous evidentiary standards (e.g. the REMARK guidelines for prognostic biomarkers, the STARD framework for diagnostic accuracy studies). The Hcp1-ICT, having been prospectively validated in >650 patients across multiple Thai provinces [14,15], is a candidate for WHO PQ submission. For next-generation multiplex devices, early engagement with regulatory agencies (FDA, CE, WHO PQ) will be essential to align study designs with prequalification requirements. Partnerships with diagnostic manufacturers, particularly regional producers in Southeast Asia and India, will be pivotal to ensure affordable pricing ($2–5 per test) and supply chain resilience. A tiered pricing model, subsidized for public health programs in low-income countries and market rate for private sector, could balance access and commercial viability, as has been successfully implemented for malaria RDTs and HIV self-tests.

## Challenging questions for the next decade

Beyond the priorities listed above, two fundamental questions merit open debate. First, should we continue searching for universal diagnostic biomarkers that work across all clinical presentations and geographic regions, or should we shift toward personalized diagnostic algorithms that incorporate patient immune status, infecting strain genotype, and clinical syndrome? The latter approach may be more feasible given the heterogeneity of melioidosis. Second, how do we distinguish serological positivity due to past exposure from active infection in highly endemic populations, even when using infection‑upregulated antigens like Hcp1? This question calls for large‑scale longitudinal cohort studies to define context‑specific diagnostic thresholds.

## Priorities for the next decade

To translate this framework from concept to impact, we propose three actionable priorities for the global melioidosis research community: (1) Establish an International Melioidosis Proteome Consortium to standardize sample collection protocols, share raw proteomic and genomic data, and conduct multi-center prospective validation of candidate biomarkers across diverse endemic populations (Southeast Asia, Australia, India, the Americas). (2) Co-develop multi-analyte POC platforms through engineering partnerships, prioritizing microfluidic or multiplexed lateral flow devices capable of simultaneously detecting Hcp1, POMCR/PPEP, and anti-GroEL IgG, with embedded machine learning algorithms for real-time risk scoring. (3) Conduct prospective implementation studies in district-level hospitals, comparing the proposed virulence-informed diagnostic algorithm against standard care using cluster-randomized designs, with primary endpoints of 28-day mortality, time-to-appropriate therapy, and cost-effectiveness. Generalizability to other intracellular pathogens. The virulence-driven translational pipeline outlined here (mechanistic prioritization, antigenic validation, POC format optimization, and field validation) is broadly applicable to other neglected tropical diseases caused by intracellular bacteria, including tuberculosis, brucellosis, leptospirosis, and melioidosis itself. For each, the key is to first identify virulence determinants specifically expressed during active human infection (e.g. ESAT-6/CFP-10 for *Mycobacterium tuberculosis*) and then adapt the same four-stage pipeline. We anticipate that this framework will serve as a replicable template for accelerating diagnostic innovation across multiple pathogens.

## Impact of bacterial adaptation on diagnostic detection

An additional consideration for virulence‑informed diagnostics is the potential for bacterial adaptation to affect target detection. *B. pseudomallei* exhibits considerable genetic and phenotypic diversity across environmental and clinical isolates, including variation in virulence gene expression [28]. Antigenic variation, phase variation, or downregulation of virulence factors during chronic or latent infection could reduce the sensitivity of antibody‑based tests targeting specific proteins such as Hcp1 or POMCR. Furthermore, immune selection pressure may drive the emergence of strains with altered epitope profiles, potentially affecting assay performance over time. While Hcp1 appears to be conserved across diverse clinical isolates based on current data [13], systematic evaluation of antigenic diversity across geographic regions is warranted. Future diagnostic development should incorporate surveillance of target antigen sequences and consider multi‑target panels to mitigate the risk of escape variants.

## Conclusion

This review delineates a promising roadmap to address the translational bottleneck in melioidosis management. Our integrated, virulence-informed framework establishes an end-to-end pipeline from computational biomarker discovery to field-deployable diagnostic tools. Three coordinated actions are paramount: establishing an international consortium to validate biomarkers across endemic populations; co-developing affordable multi-analyte POC platforms; and implementing explainable AI to ensure clinical transparency. While the framework has limitations, particularly during low-burden infection stages, it is directly applicable to other intracellular pathogens. By addressing health economics and real-world feasibility, this strategy could reduce mortality, enable risk stratification, and alleviate the global burden of melioidosis and other neglected tropical diseases.

## Supplementary Material

Supplemental Material

## Data Availability

Data sharing is not applicable to this article as no new data were created or analyzed in this study. All data supporting the findings are derived from previously published articles, as cited in the manuscript.
